# Light-Emitting-Diode-Assisted, Fungal-Pigment-Mediated Biosynthesis of Silver Nanoparticles and Their Antibacterial Activity

**DOI:** 10.3390/polym14153140

**Published:** 2022-08-01

**Authors:** Nobchulee Nuanaon, Sharad Bhatnagar, Tatsuya Motoike, Hideki Aoyagi

**Affiliations:** 1 Life Science and Bioengineering, Graduate School of Life and Environmental Sciences, University of Tsukuba, 1-1-1, Tennodai, Tsukuba 305-8572, Ibaraki, Japan; n.nobchulee@gmail.com; 2 Faculty of Life and Environmental Sciences, University of Tsukuba, 1-1-1, Tennodai, Tsukuba 305-8572, Ibaraki, Japan; sharad.bhatnagar88@gmail.com; 3 Management Headquarters, Ushio Lighting Inc., Hatchobori, Chuo-ku, Tokyo 104-0032, Japan; t-motoike@ushiolighting.co.jp

**Keywords:** antibacterial activity, light-emitting diodes LEDs, nanoparticle biosynthesis, silver nanoparticles, synergistic activity, *Talaromyces purpurogenus*

## Abstract

Nanoparticle synthesis, such as green synthesis of silver nanoparticles (AgNPs) using biogenic extracts, is affected by light, which changes the characteristics of particles. However, the effect of light-emitting diodes (LEDs) on AgNP biosynthesis using fungal pigment has not been examined. In this study, LEDs of different wavelengths were used in conjunction with *Talaromyces purpurogenus* extracellular pigment for AgNP biosynthesis. AgNPs were synthesized by mixing 10 mL of fungal pigment with AgNO_3_, followed by 24 h exposure to LEDs of different wavelengths, such as blue, green, orange, red, and infrared. All treatments increased the yield of AgNPs. The solutions exposed to blue, green, and infrared LEDs exhibited a significant increase in AgNP synthesis. All AgNPs were then synthesized to determine the optimum precursor (AgNO_3_) concentration and reaction rate. The results indicated 5 mM AgNO_3_ as the optimum precursor concentration; furthermore, AgNPs-blue LED had the highest reaction rate. Dynamic light scattering analysis, zeta potential measurement, transmission electron microscopy, and Fourier transform infrared spectroscopy were used to characterize the AgNPs. All LED-synthesized AgNPs exhibited an antimicrobial potential against *Escherichia coli* and *Staphylococcus aureus*. The combination of LED-synthesized AgNPs and the antibiotic streptomycin demonstrated a synergistic antimicrobial activity against both bacterial species.

## 1. Introduction

In the past few years, increasing application of nanotechnology has ushered a progressive uptick in the field of metal nanoparticle research [1]. Metal nanoparticles have attracted interest in various fields of application, including nanosensors [2], nanocatalysts [3], textiles [4], medicine and cancer therapeutics [5,6], wound healing [7], water treatment [8,9], plant disease control [10,11], and antimicrobials [12], owing to their distinct properties. Both physical and chemical synthesis of metal nanoparticles, including top-down and bottom-up processes, are energy-intensive and adversely affect the environment via residual contaminants [1,13]. Thus, green synthesis of metal nanoparticles using biocompatible sources, such as plant [14,15], bacterial, and fungal [16] extracts, is considered an alternative and cost-effective option for metal nanoparticle production. Biological processes provide a simpler method of synthesis, use less energy, and are environmentally friendly compared with physical and chemical processes [13]. Among various nanoparticles, silver nanoparticles (AgNPs) have attracted wide interest because of their unique properties and advantages, especially their antimicrobial activity, leading to the development of biosynthetic processes wherein different plant and microbial extracts are used as reducing, capping, and stabilizing agents [17].

Several factors are known to drive AgNPs biosynthesis, with metal salt concentration, pH, temperature, and reaction time being the most important [13]. Light has also been shown to affect the biosynthesis of AgNPs when plant and microbial extracts are used as reducing and capping agents [18,19,20]. The study of light on the synthesis of AgNPs using extracellular polymeric substances of *Chlamydomonas reinhardtii* showed that light induced the production of AgNPs [21]. Similarly, light was used to induce fungal-mediated AgNPs biosynthesis using *Penicillium oxalicum* [22] and *Pleurotus florida* [23], whereas the use of light on size and shape control to AgNPs biosynthesis has not been widely studied. Previously, the use of blue light-emitting diode (LED) as the conversion tool to nanodecahedron AgNPs using chemical synthesis was reported [24]. Moreover, LEDs at different wavelengths (405, 590, and 720 nm) have been shown to control the shape of AgNPs to dodecahedron, triangular, and rod shape respectively during chemical synthesis using I-2959 aqueous solution. This study suggested that light at a specific wavelength can induce changes to the electromagnetic fields of AgNPs, resulting in the shape conversion of particles [25]. As the use of LEDs in AgNPs synthesis has shown a promising effect in chemical synthesis, LEDs light effect on the green synthesis of AgNPs should also be studied in the presence of light-interacting biocomponents such as fungal extracellular pigment extracts acting as a reducing agent, in order to formulate an eco-friendly size and shape control strategy. Fungal extracellular pigments have been studied for the bio-generation of metal nanoparticles owing to their high protein content and secondary metabolite components; therefore, they are considered suitable bio-factories [16]. Fungal pigment extracts from *Talaromyces purpurogenus* (*T. purpurogenus*) and *Monascus* are rich in phytochemicals, have potential for industrial pigment production, and have previously exhibited antiproliferative and antioxidant activities, especially their extracellular pigments [26,27]. This high content of secondary metabolites and proteins in fungal extracellular pigment are responsible for silver salt reduction in the formation of AgNPs. Furthermore, extracellular pigment production facilitates extraction and has been previously shown to reduce silver salts, rendering the pigments suitable for nanoparticle biosynthesis. A previous report on AgNP biosynthesis using *T. purpurogenus* showed that fungal-extracellular-pigment-mediated AgNPs are light-sensitive, with light affecting their size distribution [28]. The report also indicated the possibility of light-assisted AgNP synthesis using a fungal extracellular pigment as a reducing and capping agent. Although the effect of LED light on the size and shape of biosynthesized AgNPs using *T. purpurogenus* extracellular pigments has not yet been established, there have been reports on LED light-assisted size and shape control of AgNPs using I-2959 aqueous solution [25] and salmon DNA extract combined with NaBH_4_ [29]. Based on data from recent studies, different LED wavelengths might affect fungal pigment extract-mediated AgNPs biosynthesis, which can perhaps be used as a novel technique for AgNP biosynthesis.

In this study, we propose a green AgNP biosynthesis method aided by different LED wavelengths, such as blue (450 nm), green (525 nm), orange (590 nm), red (660 nm), and infrared (850 nm), using *T. purpurogenus* fungal extracellular pigment as a reducing and capping agent. The AgNP production rate with different LEDs was observed using surface plasmon resonance (SPR) with a UV–Vis spectrophotometer. The effect of LEDs on the AgNP size, shape, distribution, and stability were examined. Functional groups related to pigment-mediated AgNP formation were also identified. Further, the antimicrobial potential of different AgNPs synthesized using LEDs against *Escherichia coli* and *Staphylococcus aureus* was determined as well as their synergistic effect in combination with antibiotic: streptomycin against both bacteria.

## 2. Materials and Methods

### 2.1. Chemicals

Sucrose, hipolypepton, yeast extract (Nihon Seiyaku, Tokyo, Japan), magnesium sulfate heptahydrate (MgSO_4_·7H_2_O), dipotassium hydrogen phosphate (K_2_HPO_4_), sodium nitrate (NaNO_3_), potassium chloride (KCl), ferrous sulfate heptahydrate (FeSO_4_·7H_2_O), ethanol (99%, special grade), and silver nitrate (AgNO_3_) were purchased from Fujifilm Wako Pure Chemical Corporation (Osaka, Japan).

### 2.2. Fungal Extracellular Pigment Production and Extraction

*T. purpurogenus* was obtained from the Cell Cultivation Laboratory (Faculty of Life and Environmental Sciences, University of Tsukuba, Tsukuba, Japan). Fungal extracellular pigment production and extraction were performed as described previously [18], with minor modifications as follows. For *T. purpurogenus* extracellular pigment production, 10 mL of spore suspension was inoculated into the inoculum medium (100 mL of yeast extract 5 g/L, sucrose 30 g/L, K_2_HPO_4_ 1 g/L, and 10 mL/L Czapek extract (NaNO_3_ 30 g/L, KCl 5 g/L, MgSO_4_·7H_2_O 5 g/L, and FeSO_4_·7H_2_O 0.2 g/L), adjusted to pH 5.0), and incubated at 30 °C with shaking at 150 rpm in the dark for 24 h. Then, 5% (*v*/*v*) of the inoculum medium was transferred to the production medium (100 mL of sucrose 50 g/L, peptone 25 g/L, K_2_HPO_4_ 2 g/L, MgSO_4_·7H_2_O 2 g/L, and 1% (*v*/*v*) of salt solution: NaNO_3_ 1 g/L, KCl 0.05 g/L, and 0.001 g/L FeSO_4_·7H_2_O, adjusted to pH 5.0), and incubated for 10 days at 30 °C and 150 rpm in the dark for red pigment production.

Extracellular pigment extraction was conducted by centrifuging 40 mL of the production medium at 6700× *g* and 4 °C (M-160-IV, SAKUMA, Tokyo, Japan) for 20 min, followed by separation of the supernatant from the biomass. The supernatant was collected and the extracellular pigment was extracted by mixing with 70% (*v*/*v*) ethanol in a 1:1 ratio at 150 rpm for 3 h. Subsequently, the mixture was evaporated using a rotary vacuum evaporator (N-1000 series, Eyela, Tokyo, Japan) to remove the ethanol and concentrate the pigment, and the extracellular pigment was filtered through a 0.45 μm filter (Advantec Toyo Kaisha, Tokyo, Japan). Finally, for AgNP biosynthesis, 1 N NaOH was used to adjust the extracellular pigment pH to pH 10.

### 2.3. Effect of Different Light Wavelengths on AgNP Biosynthesis

**Figure 1:**
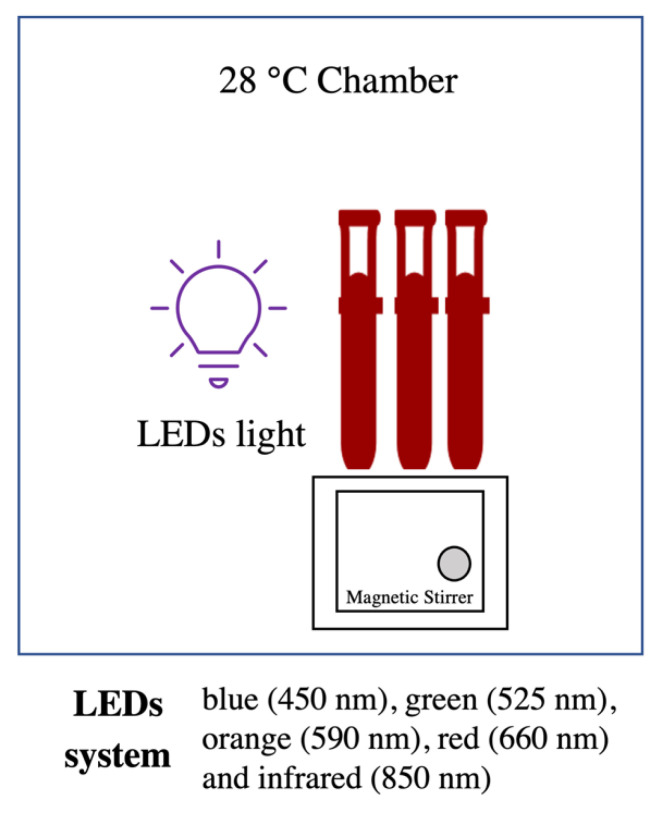
Schematic diagram of LED-assisted *T. purpurogenus* extracellular-pigment-mediated AgNP biosynthesis.

A 10 mL reaction mixture containing extracellular pigment (adjusted to pH 10) with 2 mM AgNO_3_ was prepared, with a final pigment concentration of 0.5 g/L. The reaction mixture was then kept in a chamber connected to different LED light systems with light intensity of 100 mW/cm^2^ (Advantest Optical Power Meter TQ8210, Tokyo, Japan): blue (450 nm), green (525 nm), orange (590 nm), red (660 nm), and infrared (850 nm), at 28 °C. White light and dark conditions were used as control treatments. A magnetic stirrer system was used to ensure the homogeneity of reaction mixtures (Figure 1). AgNP synthesis was evaluated by UV–Vis spectrum scanning of the samples in the range of 300–800 nm using a UV–Vis spectrophotometer (V-550, JASCO, Tokyo, Japan) at various time points over 24 h. Absorbance at a selected wavelength (412 nm) was measured at 0, 2, 4, 8, 12, and 24 h to determine the rate of AgNP synthesis using different LEDs. The colour change of the treatment mixture was visually observed to determine the AgNP synthesis.

### 2.4. Optimization of Metal Salt Concentration and Time Course Study for AgNP Biosynthesis

The LED light wavelengths selected from previous experiments were used to determine the optimum metal salt precursor concentration for AgNP synthesis. Different concentrations of AgNO_3_ (2, 5, 10, 15, and 20 mM) were used to synthesize AgNP under the aforementioned reaction conditions. The reaction mixture was then exposed to the selected LEDs in a chamber at 28 °C, and homogeneity was ensured using a magnetic stirrer for 24 h. Dark condition was used as a control treatment. UV–Vis spectrophotometry and visual confirmation were used to confirm AgNP synthesis.

The optimum metal-salt precursor concentration was used to determine the time course of the selected LED light for AgNP synthesis. AgNP synthesis was evaluated at 0, 2, 4, 8, 12, and 24 h using UV–Vis spectrophotometry (300–800 nm). Absorbance at 412 nm was used to express the AgNP yield obtained using different LEDs. Three replicates were analyzed, and data were expressed as mean ± standard error.

### 2.5. AgNP Characterization

After 24 h of synthesis, AgNPs were centrifuged at 4800× *g* (AG 22331, Eppendorf, Hamburg, Germany) for 10 min, and the resultant pellet was washed twice with deionized water. The washed pellet was resuspended in deionized water by ultrasonication (3510-DTH, Branson, CT, USA) for 10 min before characterization. Transmission electron microscopy (TEM; H-7650, Hitachi, Tokyo, Japan) was used to analyze the size and shape of the biosynthesized AgNPs. Ten microliters of AgNPs (dilution factor = 20) were dropped onto a carbon-coated formvar copper grid, and the sample was air-dried before observation at 80 kV. Dynamic light scattering (DLS; Zetasizer Nano ZS, Malvern Panalytical, Worcestershire, UK) was used to determine the size distribution and stability (zeta potential) of AgNPs. Functional groups relevant to AgNP biosynthesis were analyzed by Fourier transform infrared spectroscopy (FTIR; JASCO FT/IR-6800, Tokyo, Japan) using lyophilized AgNPs mixed with KBr pellets.

### 2.6. Antimicrobial Activity of Biosynthesized AgNPs

*E. coli* K 12 and *S. aureus* ATCC6538P obtained from the Cell Cultivation Laboratory (Faculty of Life and Environmental Sciences, University of Tsukuba, Tsukuba, Japan) were used as representative gram-negative and gram-positive bacteria, respectively, for the antimicrobial study. The overnight grown cultures of tested microbes were diluted with autoclaved double-distilled water to reach a McFarland standard of 0.5 and used to determine the antimicrobial activity of biosynthesized AgNPs.

The disk diffusion test was used for prescreening of antimicrobial activity of LED-synthesized AgNPs against both bacterial strains. The test microbes were plated on the agar plate (hipolypepton 10 g/L, yeast extract 2 g/L, and MgSO_4_·7H_2_O 1 g/L). Filter paper disks loaded with AgNPs (120 μg/disk) were placed on an inoculated agar plate, and the plates were incubated at 30 °C (*E. coli* K 12) and 37 °C (*S. aureus*) for 24 h. Zone of inhibition (ZOI) was determined by measuring the area of halo around the AgNPs impregnated disk with no visible microbial growth. The minimum inhibitory concentration (MIC) and minimum bactericidal concentration (MBC) were determined using the serial broth dilution method. A 96-well microplate (Thermo Fisher Scientific, Waltham, MA, USA) containing serial dilution of LED-synthesized AgNPs (0–500 μg/mL) was prepared. Then, the test microbes were used as the inoculum to prepare the 96-well microplate. The plates were incubated at 30 °C (*E. coli* K 12) and 37 °C (*S. aureus*) for 24 h. Streptomycin was used as the standard, whereas media without any antimicrobial agent were used as the positive control. The minimum dilution that showed no growth of tested microbe was selected as the MIC. MBC was determined by plating MIC and lower dilutions of inoculum on an agar plate with the prepared media and incubating under the previously mentioned conditions for 24 h. The highest dilution that showed no microbial growth was selected as the MBC.

Antibacterial activity of the combination of LED-synthesized AgNPs and antibiotics against both bacteria were also evaluated. AgNPs (120 μg) were loaded onto the standard disk of 10 μg streptomycin (BD Sensi-Disc Streptomycin 10, NJ, USA), and the prepared disk was placed in an inoculated agar plate. The plate was incubated at 30 °C (*E. coli* K 12) and 37 °C (*S. aureus*) for 24 h, and then the ZOI was measured. The correlation graph of the logarithmic streptomycin concentration (5–80 μg/mL) and zone of inhibition against both microbes was used to determine the activity of combined treatment, as well as individual treatments of AgNPs and streptomycin [30]. ZOIs of combined treatment and AgNPs alone were expressed in terms of ZOI of equivalent streptomycin concentration using the regression equation: y = a + b log(x), where y refers to ZOI (mm), x is the streptomycin concentration (μg/mL), and a and b are constants. The synergistic effect was determined when the effect of A + B < C, where A is the AgNPs concentration; B refers to the streptomycin concentration; and C is the combined treatment concentration, expressed in terms of corresponding equivalent streptomycin concentration, calculated from the former regression equation.

The fractional inhibitory test was determined by checkerboard titration assay using a 96-well microplate. The concentration of LED-synthesized AgNPs was 2× MIC to 1/256 MIC and that of streptomycin was 2× MIC to 1/8 MIC. The fractional inhibitory concentration (FIC) index was determined as FIC Index = (A/MICA) + (B/MICB), where A is the MIC of AgNPs in combination, B is the MIC of streptomycin in combination and MICA and MICB are the MIC of each AgNPs and streptomycin individually. The following criterion was used to determine the nature of the effect: FIC ≤ 0.5 = synergistic, FIC > 0.5–4 = additive, and FIC > 4 = antagonistic [31].

## 3. Results and Discussion

### 3.1. Effect of Different Light Wavelengths on AgNP Biosynthesis

**Figure 2:**
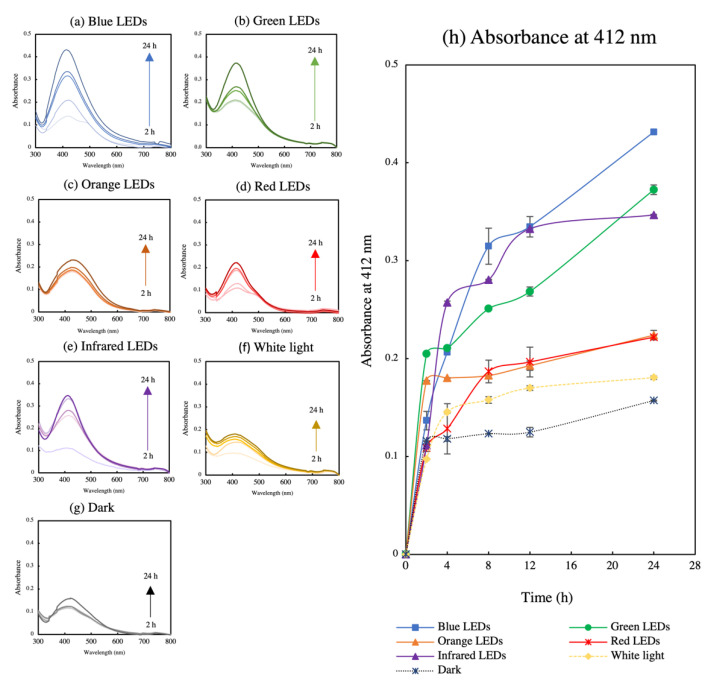
(**a**–**g**) UV–Vis spectrum of AgNPs obtained by *T. purpurogenus* extracellular pigment-mediated biosynthesis following exposure to lights at different wavelengths and (**h**) their absorbance at 412 nm for 24 h. The error bars indicate standard error (n = 3).

The effect of LEDs of different wavelengths on fungal pigment-mediated AgNP biosynthesis was confirmed by the change in colour of the reaction mixture to brown in all treatments at the 24 h mark. A change from red to brown in the reaction mixture indicated the reduction of Ag^+^ to Ag^0^ and the consequent formation of AgNPs after exposure to different LEDs [32]. A UV–Vis spectrophotometer was used to determine the SPR of the treatment mixture at an absorbance between 300 and 800 nm. Figure 2a–g shows the SPR of AgNPs biosynthesized by different LEDs. With increasing time, AgNP production was found to increase in all light treatments. The SPR band for all cases were observed between 409 and 430 nm. Different light exposure treatments revealed differences in the maximum wavelength (λ_max_) and maximum absorbance (A_max_) after 24 h, as shown in Table 1. Thus, the SPR band of all AgNPs had a wavelength shorter than 430 nm. The specific phenomenon of the SPR band with a wavelength shift near 400 nm indicated the small particle size of AgNPs. Similar results were obtained for citrate-capped AgNPs, where the decreasing SPR band signified a smaller particle size [33]. Light exposure treatments (Figure 2a–e) exhibited higher particle production than that without light (Figure 2g), indicating that light plays a crucial role in AgNP synthesis. Similarly, another report showed that LED exposure enhanced the production of biosynthesized AgNPs when fern rhizome extract was used as a reducing agent [34]. In addition, light-induced AgNP biosynthesis has previously been reported using plant [35,36,37], microbial [22,23,38], and algal [21,39,40] extracts as bioreducing agents. For AgNP formation, light or directly photoreduction excited metal salt ions mediate electron transfer to Ag^+^ and generate Ag^0^, which results in the formation of AgNPs [41]. A study on the role of light in green algal extracellular substance-mediated AgNP synthesis showed that light induced the reduction of Ag^+^ to Ag^0^, whereas algal substance complexes acted as reducing and stabilizing agents to form AgNPs [21]. Similarly, the biocomponents of *T. purpurogenus* extracellular pigment play a critical role in reducing the metal salt coupled with light-induced electron transfer in the formation of AgNPs.

Figure 2h shows the absorbance at 412 nm, representing the AgNP yield during 24 h. All light treatments exhibited an increase in AgNP production with time. After 8 h, the reaction mixture exposed to blue LED showed the highest productivity, which continued increasing with time. At the end of 24 h, the blue LED treatment demonstrated the highest AgNP yield, followed by the infrared and green LED exposure treatments. In contrast, the controls, with white light and dark conditions, showed the lowest AgNP production. The AgNO_3_ solution exposed to light in the absence of fungal extracellular pigment did not exhibit any SPR band associated with AgNPs when examined by UV–Vis spectra (Figure S1, see Supplementary Materials). Our time course study revealed the influence of blue LED on AgNP synthesis compared with that of other light exposure treatments. Different light wavelengths induced AgNP biosynthesis in a different manner owing to variation in triggering mechanisms on diverse phytochemicals present in the bio-reducing agents [29,34]. Further, AgNPs are metal nanoparticles that respond to electromagnetic (EM) fields of light [25]. Blue LED at 450 nm represent the shortest wavelength, employing the highest energy compared with the other light wavelengths in this experiment. This high energy might be the cause for excitation and rapid reduction of Ag^+^ to AgNPs in the extraction complex [34,42]. Another study has also shown that blue light irradiation enhanced AgNP biosynthesis in the presence of cherry extracts [42]. The particle size distribution determined by DLS revealed that AgNPs produced by all treatments ranged between 20 and 50 nm, except for the blue LED-irradiated AgNPs, whose size was between 2 and 15 nm (Figure S2, see Supplementary Materials). AgNPs formed by light irradiation were reported to have a size less than 60 nm [29,34], where blue light tended to increase the size distribution upon photochemical synthesis [24,25]. This work reported a smaller particle size compared with that in other previous reports of AgNPs biosynthesized in the presence of blue light [42]. TEM images revealed that the shape and appearance of AgNPs from all treatments are near-spherical. Nevertheless, a mix of non-spherical shapes was also observed in AgNPs obtained with red and orange LEDs. Previously, shape-controlled DNA-capped AgNPs were reported using LED irradiation using various combinations of DNA and NaBH_4_ concentrations [29]. The study reported that hexagonal and truncated triangle-shaped AgNPs represented the SPR band around 495–690 nm, different from that for the near-spherical shape with the SPR presented at 416–418 nm. Although our study results indicated that different shape types, such as hexagons, can possibly be obtained with red and orange LEDs, any corresponding SPR band shift might not be observed. HR-TEM performed in another study with different LED irradiation in rhizome extract AgNP biosynthesis revealed mixed AgNPs shapes with no shifts in the corresponding SPR band. All AgNPs showed an SPR band at 410–450 nm, indicating the original SPR band for the near-spherical particles [34].

### 3.2. Optimization of Precursor Concentration for AgNP Biosynthesis

**Figure 3:**
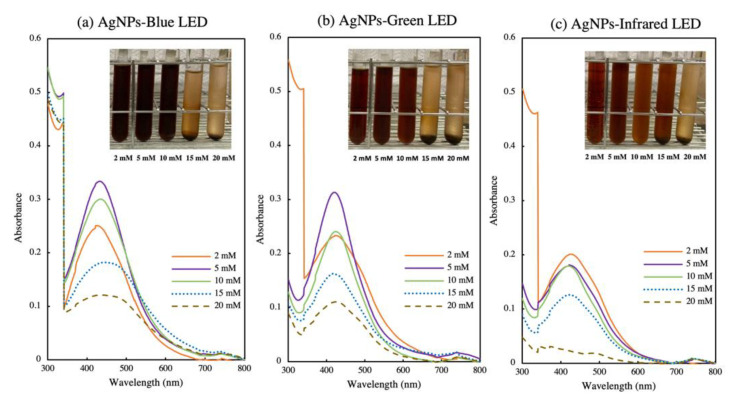
(**a**–**c**) Optimization of metal salt concentration (2–20 mM) in *T. purpurogenus* extracellular-pigment-mediated AgNP biosynthesis by blue (AgNPs-blue LED), green (AgNPs-green LED), and infrared (AgNPs-infrared LED) LEDs for 24 h.

The optimum concentration of AgNO_3_ was determined for blue (AgNPs-blue LED), green (AgNPs-green LED), and infrared (AgNPs-infrared LED) LEDs, which exhibited the highest yield among the tested wavelengths. Figure 3a–c shows the optimization of precursor concentration for AgNP biosynthesis. The SPR band showed that an increase in metal salt concentration from 2 to 5 mM resulted in higher AgNP production in the AgNPs-blue LED and AgNPs-green LED light exposure treatments, and a further increase in metal salt concentration (10–20 mM) resulted in decreased AgNP biosynthesis (Figure 3a,b). For the AgNPs-infrared LED treatment, 2 mM metal salt concentration showed the highest productivity, but as the concentration was increased to 5 mM, the production decreased (Figure 3c). An increase in metal salt concentration had previously been shown to be relevant for increasing AgNP production [43]. A higher metal salt concentration causes the greater reduction of Ag^+^ with the light-induced formation of AgNPs [34]. After 24 h, the brown colour of AgNPs was observed in all treatments (Figure 3a–c). High metal salt concentrations (15 and 20 mM) resulted in precipitation of the precursor salt under all light exposure treatments.

### 3.3. Time Course Study of AgNP Biosynthesis

**Figure 4:**
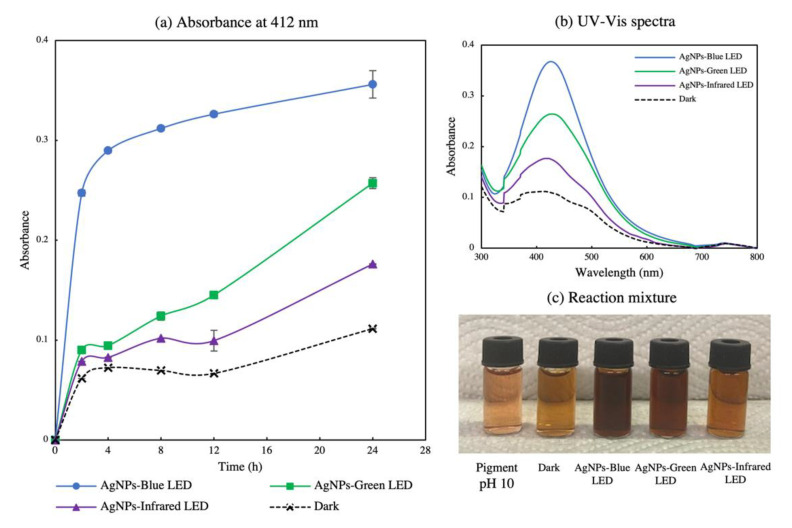
(**a**) Absorbance at 412 nm function of time for the metal salt at the optimized concentration for *T. purpurogenus* extracellular-pigment-mediated AgNPs’ biosynthesis by blue (AgNPs-blue LED s), green (AgNPs-green LED), and infrared (AgNPs-infrared LED) LEDs. The error bars indicate standard error (n = 3). (**b**) UV–Vis spectra and (**c**) image of reaction mixture upon 24 h.

An optimum concentration of 5 mM was used to conduct fungal pigment mediated AgNPs-blue LED, AgNP-green LED, and AgNPs-infrared LED biosynthesis. A time-course study at 0, 2, 4, 8, 12, and 24 h was performed to evaluate the SPR bands of the biosynthesized AgNPs. Figure 4b shows the SPR band of the biosynthesized AgNPs at 24 h. AgNPs-blue LED exhibited the highest yield with a λ_max_ at 425 nm, whereas AgNPs-green LED and AgNPs-infrared LED exhibited λ_max_ at 426 nm and 425 nm, respectively. The sharp SPR band in each treatment indicated the presence of monodispersed spherical AgNPs [25,44]. The bathochromic (red) shift of bands compared with the lower metal salt concentration revealed larger particles [45]. To demonstrate compliance with our first experiment, the effect of different light wavelengths on AgNP biosynthesis absorbance at 412 nm (Figure 4a) was considered to represent AgNP biosynthesis in the time-course study. An increase in the reaction time readily increased AgNP production [34]. AgNPs-blue LED exhibited the highest production from the beginning, followed by AgNPs-green LED and AgNPs-infrared LED. The control treatment in the absence of light showed the lowest AgNP production with respect to time. These data indicate that blue light enhances AgNP biosynthesis. Figure 4c shows the AgNP mixture after 24 h. AgNPs-blue LED presented the darkest brown colour, indicating its high AgNPs synthesis compared with that of AgNPs-green LED and AgNPs-infrared LED [35].

### 3.4. AgNP Characterization

**Figure 5:**
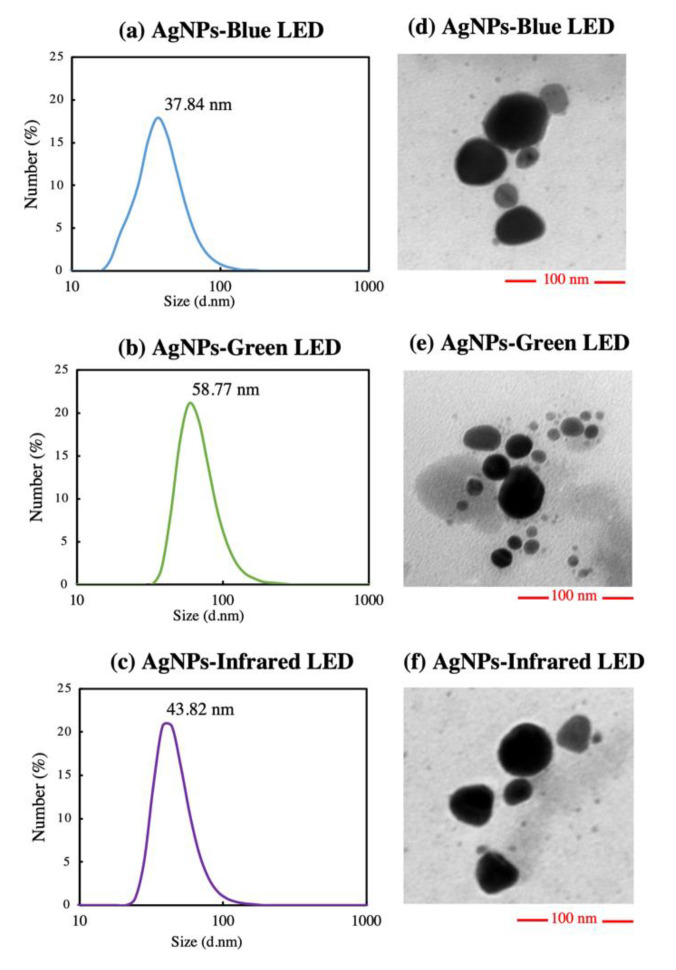
(**a**–**c**) Dynamic light scattering (DLS) analysis and (**d**–**f**) transmission electron microscopy (TEM) images of *T. purpurogenus* extracellular-pigment-mediated AgNP biosynthesis using blue (AgNPs-blue LED), green (AgNPs-green LED), and infrared (AgNPs-infrared LED) LEDs.

The size distribution of AgNPs was determined using DLS analysis. AgNPs-blue LED exhibited the smallest size distribution, as shown in Figure 5a. AgNPs-blue LED showed the maximum particle size percentage at 37.84 nm, followed by AgNPs-infrared LED and AgNPs-green LED at 43.82 and 58.87 nm, respectively (Figure 5a–c). With an increase in the precursor concentration, the SPR tended to the right, exhibiting a red shift and indicating a larger particle size [45]. However, blue-light-assisted particles retained the smallest particle size. The zeta potential was then analyzed to determine particle stability, as shown in Table 2. The zeta potential of the light-biosynthesized AgNPs showed a negative charge in all treatments. AgNPs-green LED revealed the maximum negative value, followed by AgNPs-infrared LED and AgNPs-blue LED. Stable particles reportedly have zeta potential values greater than -30mV, with a higher negative charge, showing increasing particle stability [46,47]. Figure 5d–f shows the TEM images of light-biosynthesized AgNPs. All AgNPs exhibited a near-spherical shape, with a particle size of less than 50 nm. This near-spherical shape is related to the sharp SPR band, which indicates monodispersed spherical AgNPs [44]. The DLS analysis exhibited a larger particle size compared with the TEM image because of their hydrodynamic diameter related to molecules on the surface of AgNPs [18] that moved in solution owing to Brownian motion [48,49], whereas TEM presented the size of AgNPs [50].

**Figure 6:**
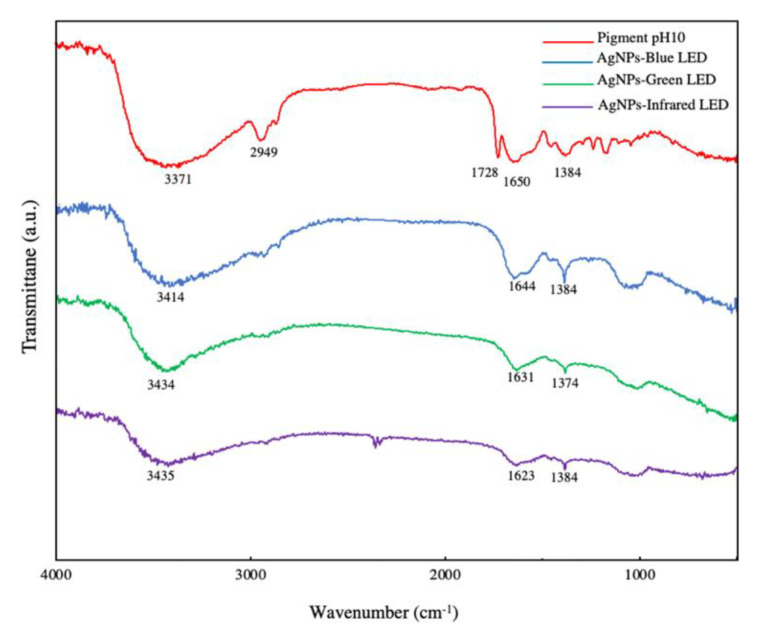
Fourier-transform infrared (FTIR) spectrum of *T. purpurogenus* extracellular pigment at pH 10 and fungus-mediated AgNPs biosynthesized using blue (AgNPs-blue LED), green (AgNPs-green LED), and infrared (AgNPs-infrared LED) LEDs.

FTIR was used to determine the functional groups present in all light-biosynthesized AgNPs and fungal extracellular pigments at pH 10. Figure 6 shows the different peaks of wavelength numbers appearing in AgNPs and the pigment at pH 10, as determined by FTIR. Table 3 describes the functional groups related to the frequency ranges found in all treatments. The results indicated that light-biosynthesized AgNPs showed no particular difference in functional groups, including the OH hydroxyl group, alkenyl NH amide, and OH–bending phenol groups. The pigment at pH 10 revealed similar functional groups present in AgNPs, but also included C–H alkanes and C=O group esters. A previous study has also reported the same vibration of functional groups present in alkaline *T. purpurogenus* extracellular pigments [18]. The presence of functional groups such as OH hydroxyl, amides, and phenol groups are related to the reduction of Ag^+^ to Ag^0^ [18,34]. *T. purpurogenus* extracellular pigment is reported to be the source of secondary metabolites, antioxidants, and protein [51]. Thus, secondary metabolites and proteins in the extracellular pigment are correlated with silver ion reduction; metal nanoparticle binding; and nanoparticle formation, capping, and stabilization [34,52]. Similarly, AgNPs derived from exposure to different LEDs showed no significant difference in their functional groups when fern rhizome extract was used for AgNP biosynthesis [34]. This research studied the biosynthesis of AgNP using *T. purpurogenus* extracellular pigment in conjugation with LED irradiation. Biocomponents present in the fungal extracellular pigment, represented by the OH hydroxyl group, amide, and phenol group of secondary metabolites, were considered to be responsible for reduction, capping, and stabilization of Ag^+^ to AgNP, with LED enhancing the electron transfer during AgNP formation, accelerating the synthesis.

### 3.5. Antimicrobial Activity

**Figure 7:**
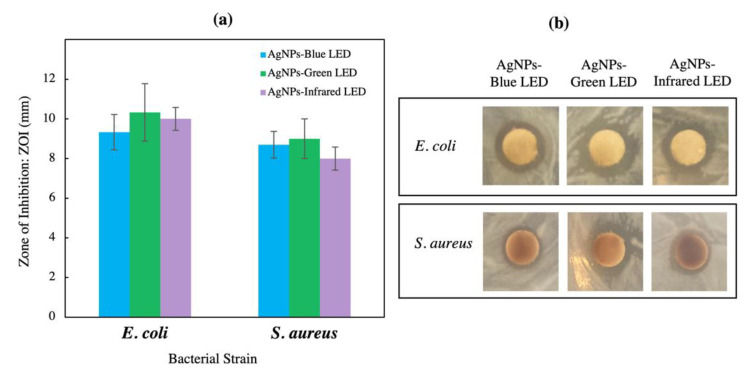
(**a**) Zone of inhibition: ZOI obtained from LED-biosynthesized AgNPs against *S. aureus* and *E. coli* K 12 evaluated by the disk diffusion method, and (**b**) images of developed ZOI. Data are expressed as the means ± SE (n = 3 replicates).

Figure 7 shows the ZOI of LED-biosynthesized AgNPs. All AgNPs exhibited a better inhibition activity against *E. coli* than *S. aureus*, with AgNPs-green LED showing higher ZOI compared with the other AgNPs. Prescreening of antimicrobial activity of AgNPs using disk diffusion showed their potential against gram-negative and gram-positive bacteria, but MIC and MBC are also required to examine the antimicrobial activity quantitatively [53,54]. Table 4 shows that the MIC and MBC values obtained for all AgNPs against *S. aureus* and *E. coli* were less than 125 μg/mL. AgNPs-green LED and AgNPs-infrared LED showed the best MICs for both pathogens, at an AgNP concentration of 62.5 μg/mL. The MBC against *S. aureus* was 125 μg/mL and was similar for all AgNPs; 62.5 μg/mL of AgNPs-green LED inhibited the growth of gram-negative bacteria such as *E. coli* compared with AgNPs-infrared LED and AgNPs-blue LED, which were required at 125 μg/mL for the same result. Thus, the disk diffusion test and MIC and MBC showed a similar trend for antimicrobial activity of biosynthesized AgNPs, but MIC and MBC required a lower concentration of AgNPs compared with the disk diffusion test. MIC and MBC determined by the broth microdilution method have been used as a common standard for antimicrobial susceptibility test recommended by Clinical & Laboratory Standards Institute (CLSI) and The European Committee on Antimicrobial Susceptibility Testing (EUCAST) [55]. The test is recommended for studying the antimicrobial ability of metal nanoparticles because reliable and quantitative results can be obtained compared with other antimicrobial activity tests such as disk diffusion or agar well diffusion [55,56]. The disk diffusion or well diffusion test is limited to non-fastidious bacteria, gives qualitative results, and is unsuitable for NPs that slowly diffuse in the agar plate [57]. The diffusion of NPs in the agar is the major hurdle in the use of these tests, thus the broth microdilution test was also employed in this research. A study on antimicrobial activity of tea leaf mediated AgNPs on gram-negative foodborne pathogens exhibited an inhibition of bacterial growth using the disk diffusion test with a small clear zone during prescreening, but in the broth microdilution, MIC and MBC showed inhibition of bacteria at lower concentrations of AgNPs [54]. AgNPs have been known for their benefits as antimicrobial agents owing to the potential of silver ions to cause damage to the bacteria cell wall, membrane, and DNA [57]. All synthesized AgNPs showed high inhibition of pathogens. As described previously, the particle sizes of all light-synthesized AgNPs ranged between 30 and 60 nm, and this small size and large surface area influenced the bactericidal action [58,59]. A similar report has revealed that fungus-mediated AgNP particle size less than 60 nm inhibited several tested bacteria, including *E. coli* [33]. The lower effect of AgNPs on the gram-positive *S. aureus* was attributed to its higher cell wall thickness compared with that of gram-negative *E. coli* [60]. Table 2 shows that AgNPs-green LED had the highest negative charge value at −47.90 ± 1.19, whereas that for AgNPs-infrared LED and AgNPs-blue LED was −44.40 ± 0.78 and −40.60 ± 1.49, respectively. Thus, the high negative charge value showed high particle stability with a direct effect on antimicrobial activity. The most stable particles, AgNPs-green LED, exhibited better antimicrobial activity against both bacteria compared with AgNPs-infrared LED and AgNPs-blue LED. Their antimicrobial potential was related to their stability, indicating that highly stabilized particles promote greater antimicrobial activity [61]. Streptomycin exhibited a stronger antimicrobial activity against both pathogens compared with all AgNPs (MIC: 15.62 μg/mL and MBC: 31.25 μg/mL for both bacteria). The agar plates depicting the MBC of biosynthesized AgNPs against *S. aureus* and *E. coli* are shown in Supplementary Figure S3 (see Supplementary Materials). A previous study on MIC and MBC using broth microdilution against *E. coli* and *S. aureus* employing chemically synthesized AgNPs showed that AgNPs inhibited both pathogens’ growth at a concentration four times higher than the antibiotic gentamicin [62]. Another study reported the MIC of bacteria-mediated AgNPs against *S. aureus* as 256 μg/mL, more than 30 times higher than the concentration of the antibiotics ampicillin (MIC 1 μg/mL), kanamycin (MIC 8 μg/mL), and tetracycline (MIC 4 μg/mL). Moreover, the antimicrobial activity against *Bacillius subtilis* was reported to be more than 100 times higher than that of antibiotics [63]. Thus, the results from the current research and literature indicate that using AgNPs could inhibit microbes, but requires a higher concentration compared with antibiotics.

**Figure 8:**
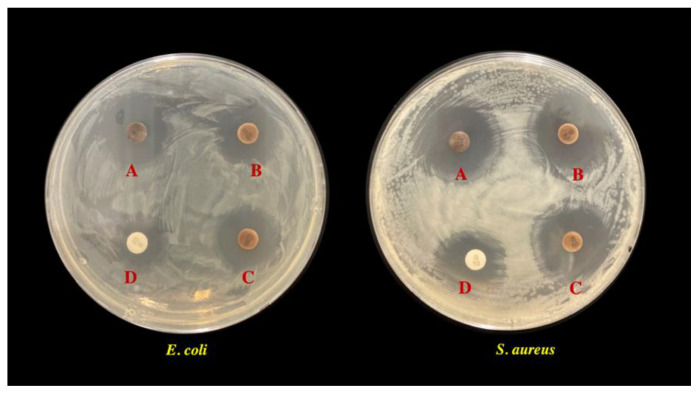
Effect of combination of LED-biosynthesized AgNPs and streptomycin against *S. aureus* and *E. coli* K 12 evaluated by the disk diffusion method: A is AgNPs-blue LED and streptomycin, B is AgNPs-green LED and streptomycin, C is AgNPs-infrared LED and streptomycin, and D is streptomycin alone. The diameter of the petri dish is 9 cm.

The combination of AgNPs and antibiotic (streptomycin) treatment against *E. coli* and *S. aureus* were evaluated by the disk diffusion test using a standard streptomycin disk loaded with LED-biosynthesized AgNPs. All combined treatments showed a synergistic effect on the antimicrobial activity against both bacterial strains compared with streptomycin alone (Figure 8). The combined treatments of AgNPs and streptomycin showed a ZOI of more than 19 mm, whereas streptomycin alone showed a ZOI at 12 ± 2.33 mm and 11.7 ± 1.67 mm against *E. coli* and *S. aureus*, respectively (Table 5). All combined treatments of AgNPs and streptomycin exhibited a slightly larger ZOI in *S. aureus* compared with *E. coli.* Previously, AgNPs and antibiotics alone showed a lower antimicrobial inhibition against gram-positive bacteria compared with gram-negative bacteria; therefore, this result demonstrated that combined treatments enhanced their antimicrobial activity against bacterial cells, especially gram-positive bacteria, *S. aureus*. Generally, AgNPs alone find it difficult to attach to the thick layer cell wall of gram-positive bacteria, but the conjugation of antibiotics with AgNPs might have promoted the bactericidal agent uptake to cells [64]. Moreover, an increase in the antimicrobial activity of the combined treatment might differ with the type of antibiotic, AgNPs, and the target organism [63,64]. The correlation graph of the logarithmic streptomycin concentration and zone of inhibition against both microbes (Figure S4, see Supplementary Materials) were used to obtain a linear relationship and regression equations, y = 9.3446x + 3.6364 (R^2^ = 0.98) and y = 10.464x + 0.2859 (R^2^ = 0.99), which were used to determine the effect of individual AgNP treatments and combined treatments in terms of equivalent streptomycin concentration against *E. coli* and *S. aureus*, respectively. The evaluation of combined treatment of AgNPs and streptomycin and the sum of individual treatments in terms of equivalent streptomycin concentration is shown in Table 6. All combination treatments exhibited a synergistic effect against both bacterial species. The comparison of combined treatment of AgNPs and streptomycin in terms of equivalent streptomycin concentration indicated that the combination exhibited a stronger activity than the sum of their individual parts, indicating a synergy in their action. A previous study showed that combination treatment of fungus *Trichoderma viride* mediated AgNPs and ampicillin significantly increased the ZOI of *E. coli* and *S. aureus* by up to 70-fold compared with AgNPs and antibiotic alone [64]. Similarly, the study of plant-meditates synthesis AgNPs in conjugation with two types of antibiotics, kanamycin and rifamycin, could increase the ZOI of studied bacterial strains including *B. cereus*, *E. coli*, *S. aureus*, and others [65].

The fractional inhibitory concentration test is shown in Table 7. All combination treatments exhibited an FIC index lower than 0.5, indicating their synergistic effect against both *E. coli* and *S. aureus*, in good correlation with the previous disk diffusion assay. The small value of the FIC index showed that the effectiveness of the combined treatments of LED-synthesized AgNPs and streptomycin on the inhibition of bacterial growth of both gram-positive and gram-negative bacteria was better than an antibiotic or LED-biosynthesized AgNPs alone. A previous report on antibiotics polymyxin B and rifampicin in combination with AgNPs showed better antibacterial activity against *Acinetobacter baumannii*, a multidrug-resistant (MDR) bacterial strain, with an FIC index less than 0.5, indicating a synergistic effect of the combined treatment of AgNPs and antibiotics [31]. AgNPs plus antibiotics also showed a significant increase in antibacterial activity against several pathogens compared with AgNPs or antibiotics alone [66], showing the synergistic potential of AgNPs and antibiotics. The synergistic effect of AgNPs combined with antibiotics owes their increased antimicrobial activity to their diverse mechanisms of microbial inhibition [64,67]. An antibiotic like streptomycin causes interruption of the ribosome formation cycle, as well as inhibition and disruption of proteins synthesis in the bacterial cell [68,69,70], whereas AgNPs possess multiple mechanisms of action against microorganisms. The presence of Ag^+^ causes cell membrane damage, and the accumulation of Ag^+^ leads to the production of reactive oxygen species (ROS), causing ATP inhibition, membrane leakage, and DNA disruption [71]. The cells suffering from AgNP toxicity exhibit a depletion in oxidative stress defense, including glutathione (GSH) reduction, superoxide dismutase (SOD), and catalase (CAT) enzyme denaturation. The small size of NPs makes it easier for them to pass through the bacterial cell wall, and consequently leads to an increase in antibiotic uptake into the cell [66,67,68,72]. Moreover, the high surface-area-to-volume ratio of AgNPs benefits the antibiotic binding and promotes their penetrating ability against the cell membrane, leading to an easier delivery to the target site of disruption [64,68,73].

## 4. Conclusions

Simple biosynthesis of *T. purpurogenus* extracellular-pigment-mediated AgNPs was performed using different LED wavelengths. AgNP production varied with the type of LED exposure, wherein blue, green, and infrared LEDs enhanced the biosynthesized AgNP yields compared with the other light sources. All of the light-synthesized AgNPs showed the dominance of near-spherical particles, whereas red and orange LEDs also exhibited the possibility of non-spherical shape induction. The optimum concentration of the metal salt precursor was 5 mM. A time course study on the biosynthesis of AgNPs-blue LED, AgNPs-green LED, and AgNPs-infrared LED was performed. Characterization studies revealed the presence of a near-spherical shape particles with sizes ranging from 30 to 60 nm, with AgNPs-blue LED exhibiting the smallest size. All AgNPs exhibited a zeta potential of more than −30 mV and showed good stability, with AgNPs-green LED showing the highest values of zeta potential, followed by AgNPs-infrared LED, and AgNPs-blue LED. Furthermore, AgNPs showed good inhibitory activity against gram-positive *S. aureus* and gram-negative *E. coli* bacteria, and AgNPs-green LED exhibited better activity than AgNPs-infrared LED and AgNPs-blue LED. The combination of all LED-synthesized AgNPs and an antibiotic, streptomycin, exhibited a synergistic effect on antimicrobial activity against both gram-positive and gram-negative bacteria. Thus, blue, green, and infrared LED-assisted rapid biosynthesis of small-sized, highly stable AgNPs possessing antimicrobial action was accomplished. However, this study showed a limited effect of LED irradiation on shape induction during AgNP biosynthesis. In the future, the synergistic effect of LEDs and different factors, including the biological extract concentration, temperature, and light intensity, on the biosynthesis of AgNPs should be examined to improve particle quality, especially shape induction. Furthermore, the effect of LEDs on AgNPs biosynthesized using different bio-reducing agents, such as plant extracts, needs to be examined.

## Figures and Tables

**Table 1:** The maximum wavelength (λ_max_) and maximum absorbance (*A*_max_) after 24 h during *T. purpurogenus* extracellular-pigment-mediated AgNPs biosynthesis by light exposure at different wavelengths.

Light Source	Maximum Wavelength $\left(\lambda_{\max}\right)$	Maximum Absorbance (*A*_max_)
Blue LEDs	413 nm	0.432 ± 0.010
Green LEDs	415 nm	0.373 ± 0.001
Orange LEDs	430 nm	0.231 ± 0.003
Red LEDs	415 nm	0.222 ± 0.002
Infrared LEDs	411 nm	0.347 ± 0.001
White light	409 nm	0.180 ± 0.001
Dark	415 nm	0.157 ± 0.002

Data are expressed as the means ± SE (n = 3 replicates).

**Table 2:** Zeta potential of LED-assisted biosynthesized AgNPs.

Biosynthesized AgNPs	Zeta Potential (mV)
AgNPs-Blue LED	−40.60 ± 1.49
AgNPs-Green LED	−47.90 ± 1.19
AgNPs-Infrared LED	−44.40 ± 0.78

Data are expressed as the means ± SE (n = 3 replicates).

**Table 3:** Frequency range obtained by FTIR and the corresponding functional groups.

Frequency Range (cm^−1^)	Functional Group	Treatment			
		Pigment pH 10	AgNPs-Blue LED	AgNPs-Green LED	AgNPs-Infrared LED
3570–3200	H, OH, hydroxyl group	+	+	+	+
3000–2840	C–H alkane	+	-	-	-
1730–1715	C=O group ester	+	-	-	-
1650–1600	NH amide group	+	+	+	+
1390–1310	OH bending phenol	+	+	+	+

(+): functional group is present, (-): functional group is not present in the treatment.

**Table 4:** Antimicrobial activity of LED-biosynthesized AgNPs against *S. aureus* and *E. coli* K 12 evaluated by the minimum inhibitory concentration: MIC and the minimum bactericidal concentration: MBC (μg/mL).

Bacterial Strain	Antimicrobial Treatment							
	AgNPs-Blue LED		AgNPs-Green LED		AgNPs-Infrared LED		Streptomycin	
	MIC	MBC	MIC	MBC	MIC	MBC	MIC	MBC
*E. coli*	125	125	62.50	62.50	62.50	125	15.62	31.25
*S. aureus*	125	125	62.50	125	62.50	125	15.62	31.25

**Table 5:** Zone of inhibition obtained from the combination of LED-biosynthesized AgNPs and streptomycin against *S. aureus* and *E. coli* K 12 was evaluated by the disk diffusion method.

Bacterial Strain	Zone of Inhibition (mm)						
	Individual Treatment				Combined Treatment with Streptomycin		
	AgNPs-Blue LED	AgNPs-Green LED	AgNPs- Infrared LED	Streptomycin	AgNPs- Blue LED	AgNPs-Green LED	AgNPs- Infrared LED
*E. coli*	9 ± 0.89	10 ± 1.45	10 ± 0.58	12 ± 2.33	19 ± 0.67	20 ± 1.26	19 ± 0.67
*S. aureus*	9 ± 0.67	9 ± 1.0	8 ± 0.58	11 ± 1.67	20 ± 0.67	21 ± 0.58	21 ± 0.33

Data are expressed as the means ± SE (n = 3 replicates).

**Table 6:** The evaluation of combined treatment with AgNPs and streptomycin and the sum of individual treatments expressed as equivalent streptomycin concentration calculated by the regression equation.

Bacterial Strain	Equivalent Streptomycin Concentration (μg/mL)					
	Sum of Individual Treatments (A + B)			Combined Treatment with Streptomycin (C)		
	AgNPs- Blue LED	AgNPs- Green LED	AgNPs- Infrared LED	AgNPs-Blue LED	AgNPs-Green LED	AgNPs- Infrared LED
*E. coli*	15 ± 2.43	16 ± 4.19	15 ± 2.59	62 ± 5.25	89 ± 17.89	62 ± 5.25
*S. aureus*	21 ± 5.23	22 ± 5.97	21 ± 5.22	97 ± 12.24	141 ± 21.90	97 ± 12.24

A + B < C = synergistic; A = AgNPs’ concentration, B = streptomycin concentration, and C = combined treatment concentration. Data are expressed as the means ± SE (n = 3 replicates).

**Table 7:** Fractional inhibitory concentration (FIC) index of AgNPs and streptomycin combination treatments against *E. coli* and *S. aureus*.

Combined Treatment with Streptomycin	Bacterial Strains			
	*E. coli*		*S. aureus*	
	FIC Index	Nature of Interaction	FIC Index	Nature of Interaction
AgNPs-Blue LED	0.26 ± 0.04	Synergistic	0.25 ± 0.04	Synergistic
AgNPs-Green LED	0.24 ± 0.05	Synergistic	0.22 ± 0.05	Synergistic
AgNPs-Infrared LED	0.38	Synergistic	0.26 ± 0.01	Synergistic

FIC ≤ 0.5 = synergistic, FIC > 0.5–4 = additive, FIC > 4 = antagonistic. Data are expressed as the means ± SE (n = 3 replicates).

## Data Availability

The data presented in this study are available upon request from the corresponding authors.

## Supplementary Materials

The following supporting information can be downloaded at https://www.mdpi.com/article/10.3390/polym14153140/s1. Supplementary Figure S1: UV–Vis spectrum of AgNO_3_ in the absence of *T. purpurogenus* extracellular pigment exposed to different LEDs at 24 h. Supplementary Figure S2: Dynamic light scattering (DLS) and transmission electron microscopy (TEM) analysis of *T. purpurogenus* extracellular-pigment-mediated AgNP biosynthesis by light exposure at different wavelengths for 24 h. Supplementary Figure S3: Minimum bactericidal concentration (MBC) of biosynthesized AgNPs and streptomycin against *S. aureus* and *E. coli.* Supplementary Figure S4: Correlation graph of the logarithm streptomycin concentration and zone of inhibition against *E. coli* and *S. aureus*. Data are expressed as the means ± SE (n = 3 replicates).
