# Supplementary material for: Light-Emitting-Diode-Assisted, Fungal-Pigment-Mediated Biosynthesis of Silver Nanoparticles and Their Antibacterial Activity
Source: Polymers (Basel). 2022 Aug 1;14(15):3140. doi: 10.3390/polym14153140 (PMC9370687; doi:10.3390/polym14153140)
Supplement: Supplementary file 1 [file polymers-14-03140-s001.zip › polymers-1747689 supplementary.pdf]

Supplementary Materials: Light-Emitting-Diode-Assisted, Fungal-Pigment-Mediated Biosynthesis of Silver Nanoparticles and Their Antibacterial Activity

Nobchulee Nuanaon [1], Sharad Bhatnagar [2], Tatsuya Motoike [3], and Hideki Aoyagi [1,2,*]

[1] Life Science and Bioengineering, Graduate School of Life and Environmental Sciences,
University of Tsukuba, 1-1-1, Tennodai, Tsukuba 305-8572, Ibaraki, Japan; n.nobchulee@gmail.com

[2] Faculty of Life and Environmental Sciences, University of Tsukuba, 1-1-1, Tennodai, Tsukuba 305-8572, Ibaraki, Japan; sharad.bhatnagar88@gmail.com

[3] Management Headquarters, Ushio Lighting Inc., Hatchobori, Chuo-ku, Tokyo 104-0032, Japan; t-motoike@ushiolighting.co.jp

* Correspondence: aoyagi.hideki.ge@u.tsukuba.ac.jp

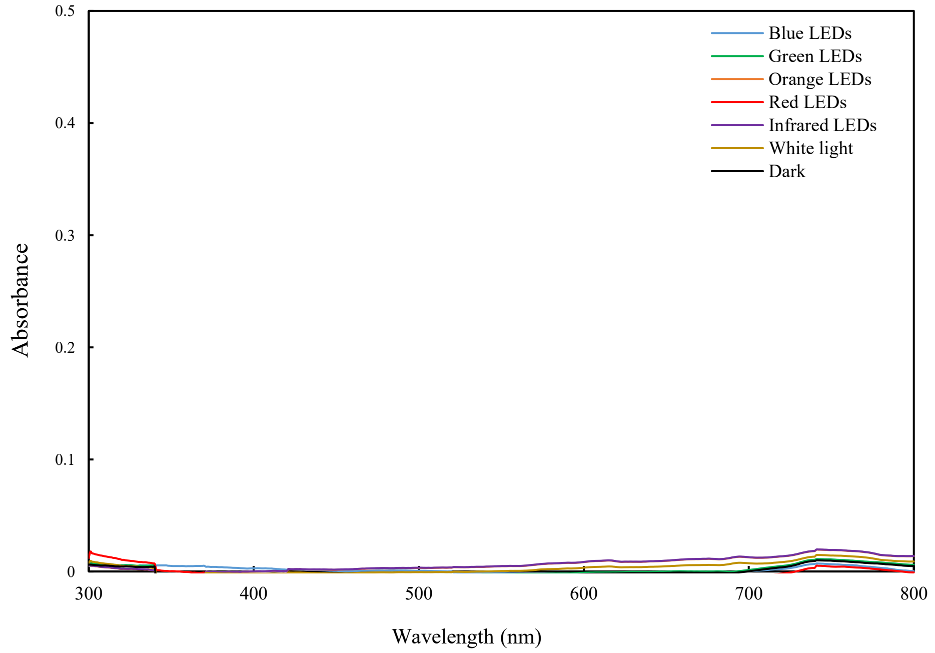

Figure S1. UV–vis spectrum of $AgNO_3$ in the absence of *T. purpurogenus* extracellular pigment exposed to different LEDs light at 24 h.

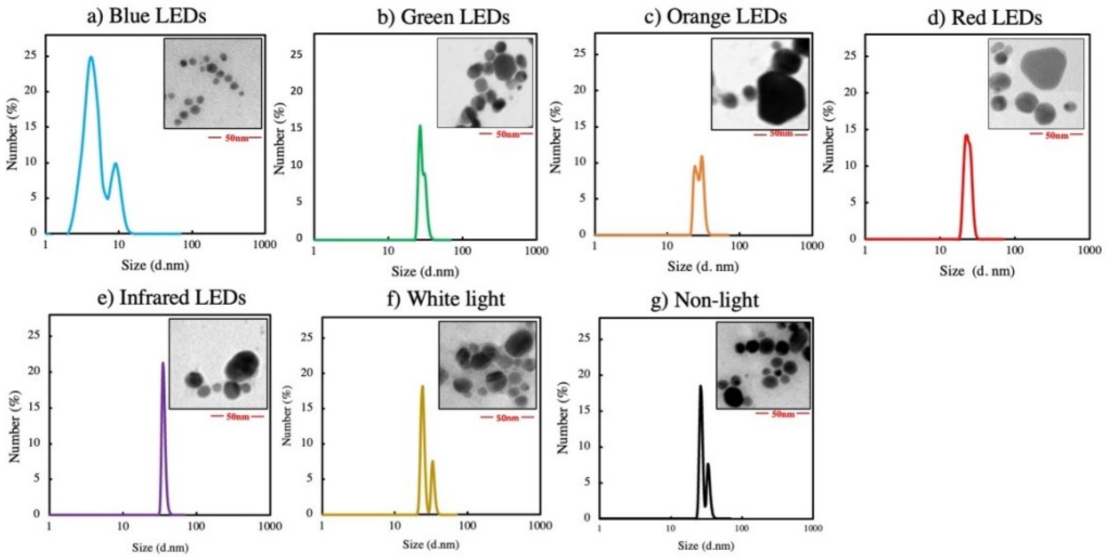

Figure S2. (**a**–**g**) Dynamic light scattering (DLS) and transmission electron microscopy (TEM) analysis of *T. purpurogenus* extracellular pigment-mediated AgNP biosynthesis by light exposure at different wavelengths for 24 h.

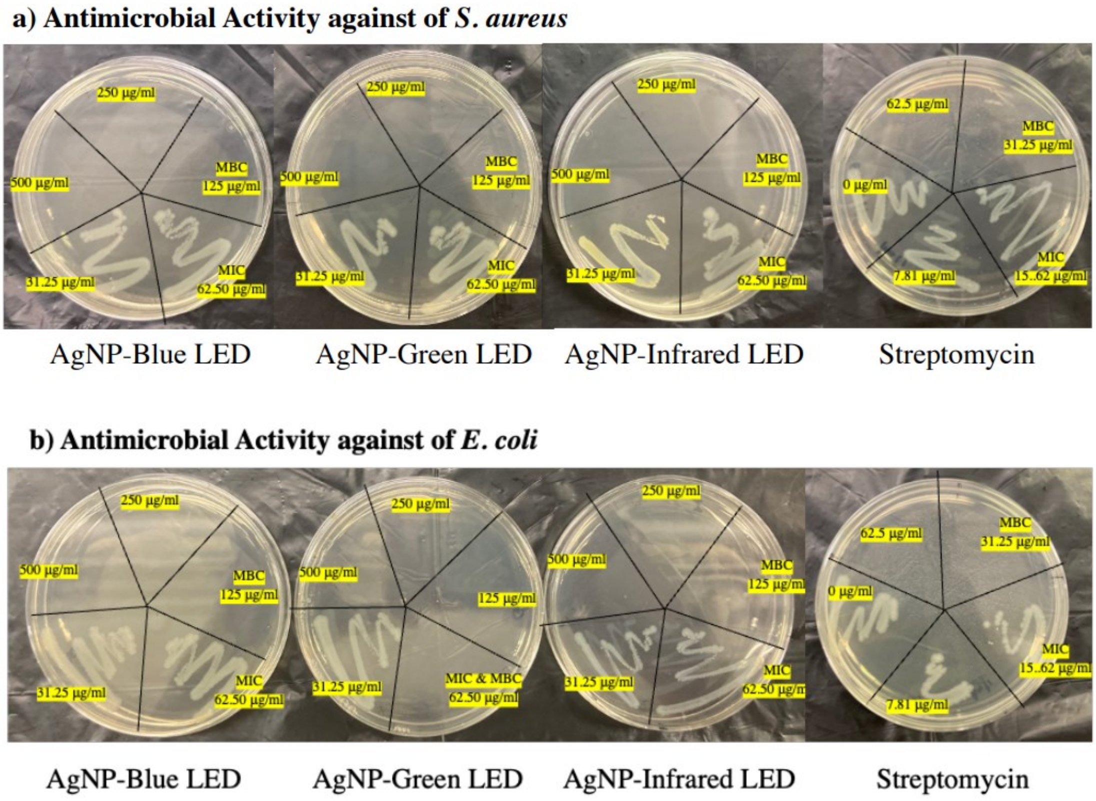

Figure S3. Minimum bactericidal concentration (MBC) of biosynthesized AgNPs and streptomycin against (**a**) *S. aureus* and (**b**) *E. coli*.

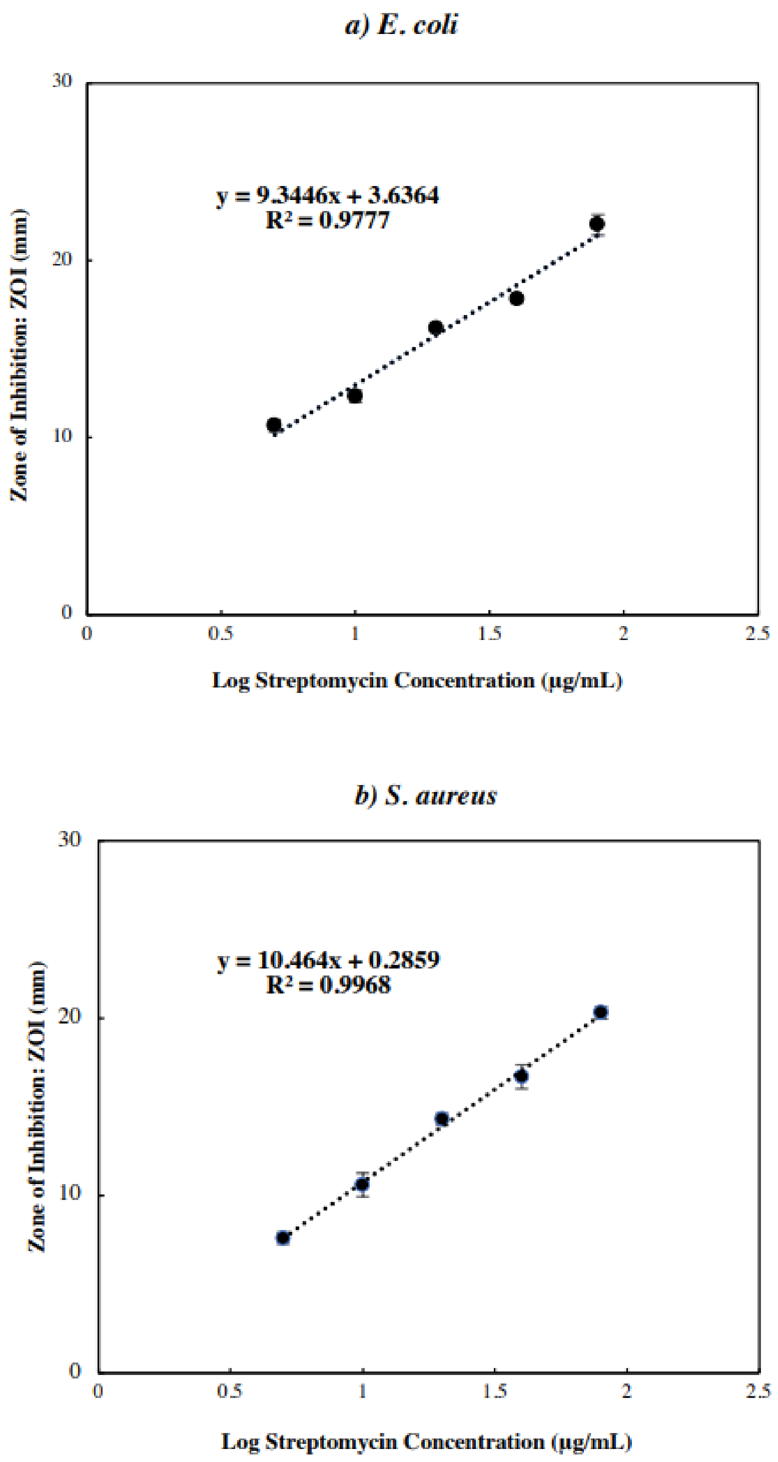

Figure S4. Correlation graph of logarithm streptomycin concentration and zone of inhibition against (**a**) *E. coli* and (**b**) *S. aureus*. Data are expressed as the means ±SE (n = 3 replicates).
